# The Gender Difference in Association between Home-Based Environment and Different Physical Behaviors of Chinese Adolescents

**DOI:** 10.3390/ijerph17218120

**Published:** 2020-11-03

**Authors:** Xiao Hou, Jing-Min Liu, Zheng-Yan Tang, Bing Ruan, Xu-Yao Cao

**Affiliations:** 1 Department of Sports Science and Physical Education, Tsinghua University, Beijing 100084, China; houxiao18@mails.tsinghua.edu.cn (X.H.); tangzy19@mails.tsinghua.edu.cn (Z.-Y.T.); cao-xy17@mails.tsinghua.edu.cn (X.-Y.C.); 2 School of Sports Medicine and Physical Therapy, Beijing Sport University, Beijing 100084, China; ruanbing@bsu.edu.cn

**Keywords:** home-based environment, adolescents, physical behaviors, physical activity, accelerometer

## Abstract

Purpose: The aim of this study was to evaluate the home-based physical activity (PA) environmental characteristics, and different types of physical behavior level of adolescents in different genders, and explore the impact of different domains of home-based PA environmental factors on different physical behaviors of adolescents in different genders. Methods: Five hundred forty-four adolescents aged from 12 to 18 years old (males: *n* = 358, females: *n* = 186) and their parents were analyzed in this cross-sectional survey. The volume of various physical behaviors of all adolescent subjects were measured by the ActiGraph wGT3X-BT accelerometer, and the level in different domains of home-based environmental characteristics were assessed by the Gattshall’s home-based PA environment questionnaire, which was answered by adolescents’ parents. The difference in the volume of different physical behaviors was examined using Kruskal–Wallis analysis. The difference in home physical environment and home social environment for adolescents was examined using one-way analysis of variance (ANOVA). Multiple linear regression analysis in the adjusted model was used to evaluate the influence of different home-based PA environmental domains (PA availability, PA accessibility, Parental role-modeling of PA, and Parental policies around PA) on different physical behaviors (sedentary behavior, SB; light-intensity physical activity, LPA; and moderate-vigorous physical activity, MVPA) of adolescents (boys and girls). Results: The volume of LPA and MVPA, the score of PA accessibility in the home physical environment, and the score of home social environment of boys are significantly higher than those of girls, while the SB volume of boys is significantly lower than that of girls. The PA availability, the parents’ role-modeling of PA in same-sex parent–child dyads, and the parents’ policies around PA in opposite-sex parent–child dyads are significantly associated with adolescents’ decreased SB and increased LPA and MVPA. Conclusion: There is significant gender difference in adolescents’ physical behaviors and home-based environmental characteristics, as well as in the association between adolescents’ physical behaviors and their home-based environment. The PA availability, the parents’ role-modeling of PA in same-sex parent–child dyads, and the parents’ policies around PA in opposite-sex parent–child dyads can significantly promote adolescents’ healthy physical behaviors.

## 1. Introduction

In recent years, insufficient physical activity (PA) and high level of sedentary behavior (SB) of adolescents has become a common public health problem all over the world, which attracts more and more researchers’ attention. It has been proved that the lack of PA for young people can cause a series of health problems, such as obesity [1], the decline in physical fitness [2,3], the increased risk of metabolic diseases [4,5,6], and even the occurrence of psychological disorders [7,8]. Therefore, it is vital to study the influential factors of adolescents’ PA and establish specific countermeasures to improve the current situation of insufficient PA of adolescents.

Many studies attribute inadequate PA of teenagers to the school-relevant PA environment and strategies [9,10], often ignoring the important role of home-based PA environment in the process of influencing adolescents’ healthy behaviors. Except school, home is where adolescents spend the most time in daily life [11,12] and the off-school PA volume of most adolescents is accumulated at home [13,14,15]. The home is the most familiar physical and social environment for individuals since they were born, and parents are their first teacher in the whole life, hence, home-based environment can exactly affect adolescents’ cognitions and physical behaviors. There are several studies indicating that the difference of PA and sports participation in adults is mainly formed in the youth period, especially via cultures transmitted through families, and this difference has lifelong continuities in many people’s physical activities [16,17]. The characteristics of the home-based environment have a significant correlation with adolescents’ weight [18], PA level, and SB [19]. Although the social ecological model involved personal, friend, home, school, and neighborhood environment is usually considered as the comprehensive influential factor to explain adolescents’ PA [16,18], one study revealed home-based environmental characteristics can produce more variance in teenagers’ moderate-vigorous-intensity PA (MVPA) than school and neighborhood environments [19].

Home-based environment consists of home physical environment and home social environment. The home physical environment includes the areas both inside (e.g., sports equipment at home) and outside (e.g., yard) that can promote or hinder adolescents’ PA [20], while the home social environment involves the role of family members on adolescents’ PA, especially parents (e.g., parents’ PA level, logistical support, and behavior encouragements) [21]. Most researchers only focus more on the effect of home-based social environment (e.g., parents’ support of adolescents’ PA) and find that parents’ own PA level, their role-modeling, and PA encouragements or strategies have a continuous positive correlation with the PA level of their children [22,23,24,25,26,27,28]. Specifically, Xu et al. find that the teenagers whose parents often take their teenagers to places where she/he can be physically active and encourage their children to exercise or play outside will have higher PA levels [29]. However, due to the lack of consistent conclusion and uniform division criteria about home physical environment, less is known about the effect of home physical environment on adolescents’ PA [22]. For example, Patnode et al. demonstrate that the number of small sports facilities at home can be used to predict boys’ MVPA level [30]. Nevertheless, Dunton et al. indicate the physical activity of girls is significantly related to availability and diversity of sports equipment [31].

In this study, we plan to conduct a comprehensive evaluation of adolescents’ home-based PA environment (both home physical environment and home social environment), demonstrate the home-based PA environmental characteristics and different types of physical behavior level of adolescents in different gender, and explore the impact of different domains of home-based PA environmental factors on different physical behaviors (SB, light-intensity physical activity (LPA), and MVPA) of adolescents in different gender.

## 2. Materials and Methods

### 2.1. Study Population

A cross-sectional health survey design was used in this study. The authors assumed a median effect size at 0.25, alpha level at 0.05, and power at 0.8, and calculated a sample size of 269 using G*power software (version 3.1.9.7, The G*Power Team, Düsseldorf, Germany). In consideration of the drop out of participants and inclusion and exclusion criteria, the sample size was determined as 580. A total of 580 adolescents aged from 12 to 18 years and their parents were recruited on 15 May 2019 and the data collection started from 20 May 2019 to 18 June 2019. Due to the exclusions based on the accelerometer wear time and nonresponse subjects, the data of 544 subjects were included in this study finally; there were 358 boys (15.38 ± 1.62 years; range: 12–18 years) and 186 girls (14.41 ± 3.11 years; range: 12–18 years). Inclusion criteria were (1) aged from 12 to 18, (2) absence of physical disability or severe diseases that hinder PA, and (3) nonsingle parent family (because this study does not explore the effect of family structure on adolescents’ PA).

### 2.2. Measurements and Instruments

The recorded sociodemographic information in this survey included age (years), gender, height (cm), body mass (kg), parental education, number of siblings, and family monthly outcome (RMB), which were measured by questionnaire. The results are shown in Table 1. The outcome included the home-based PA environment characteristics investigated through a questionnaire answered by the adolescents’ parents and the weekly PA of adolescents measured by ActiGraph wGT3X-BT (ActiGraph, Pensacola, FL, USA) accelerometer.

For the measurement of home-based PA environment characteristics, we used Gattshall’s home-based PA environment questionnaire [32]. Based on Golan’s model of family-related environmental influence [33], Gattshall et al. constructed a conceptual model of home-based environmental influence on adolescents’ PA and eating behavior, and then developed a questionnaire with acceptable reliability and validity, which was used to evaluate the characteristics of home environment [32]. This questionnaire measured home-based PA environmental characteristics in four domains: PA availability, PA accessibility (Cronbach’s α = 0.66), Parental role-modeling of PA (Cronbach’s α = 0.68), and Parental policies around PA (Cronbach’s α = 0.79). The former two domains were the home physical environment, and the latter two domains were the home social environment. Among them, “PA availability” scale included 22 items that investigated the availability of PA equipment and sites at or around home. Parents were required to answer these items and record the score as 0 or 1, according to the fact whether there were these facilities at or around home. The total score of these 22 items was used to assess “PA availability” of this home. Based on the common sports items that Chinese adolescents usually used, we revised “sandbox” and “hockey equipment” into “dance room, yoga room, Taekwondo room” and “sports application (APP) in digital equipment,” respectively. The “PA accessibility” domain, the “Parental role-modeling of PA” domain, and the “Parental policies around PA” domain were scored by Likert’s five-point rating method (0–4) with a higher score representing a more positive response (i.e., the “PA accessibility” were rated as “0” to “more than 4”, the “Parental role-modeling of PA” and the “Parental policies around PA” were rated as “never” to “frequently”). In addition, if the questions involved a negative impact on the home-based PA environment, the items were scored reversely. For these three domains, the average score of all the items in each domain scale was their final score. Furthermore, for the scale of home social environment, both father and mother should be investigated separately. The specific items of each home-based PA environment domain are presented in Appendix A.

The volume of different physical behaviors of adolescents was measured by an ActiGraph wGT3X-BT accelerometer with a sampling frequency of 100 Hz [34]. The ActiGraph accelerometer attached to a soft elastic belt was located on the right midaxilla line at the level of one’s iliac crest. The adolescent subjects were informed of the wearing requirements: they must wear the accelerometer at the correct position for seven consecutive days except for periods of swimming and bathing. The seven consecutive testing days were a regular seven-day school week, which included five school days and the complete weekend with two days. In addition, the PA intensity of adolescents was graded by the cut points (unit: counts per minute, CPM) of ActiGraph accelerometer based on the MET (metabolic equivalent, the MET is defined as a unit of PA intensity) formula:

(1) METs = 2.757 + (0.0015 × CPM) − (0.08957 × age) − (0.000038 × CPM × age)

with assumed MET thresholds of 3, 6, and 9 METs, which represented the threshold of low, moderate, and vigorous PA intensity, respectively [35]. The ActiLife v6.13.3 software was applied to initialize, download, and calculate PA data. Based on the cut point set in ActLife, all the values are for 60 s epochs. Nonwear period, defined as ≥90 consecutive minutes of 0 counts, was removed by the software [36], and a minimum for total wear time with recorded data in the accelerometer was defined as 10 h a day on 4 days.

### 2.3. Procedures

The students and parents were approached and collected at school together through a “School Sports Open Day” by the colleagues in the adolescent study project from Tsinghua University’s Department of Sport Science, Beijing, China.

When young subjects and their parents agreed to participate in this study, they received a questionnaire of specific information about illness, physical state, or disability to verify whether they met the inclusion criteria of this study. After providing information about the study (purpose, expected time and procedure of the questionnaire interview, and PA measuring steps), an informed consent was signed.

The entire test process included two parts. The first part was that adolescents’ parents (both father and mother) should answer the Gattshall’s home-based PA environment questionnaire, and the second part was a survey of sociodemographic characteristics and the quantitative measurement of weekly volume of different physical behaviors using the ActiGraph wGT3X-BT accelerometer. These two parts were finished in one regular seven-day school week. In the whole study procedure, the project colleagues, the head teacher, and physical education teacher of the recruited students supervised and reminded students to wear the accelerometer.

### 2.4. Ethical Considerations

All participants had detailed procedures introduced to them before participating in the study and then signed the informed consent documents. Participants were also clearly informed that they could withdraw from the study at any time for any reason. The results of any participant’s own PA record in this study were confidential, and these results could be included in every adolescent’s weekly PA report and then provided to each participant. The authors declared that all the experiments of this study complied with the current laws of China in which they were performed. The study was approved by the medical ethics committee of Tsinghua University (project number: 20190095).

### 2.5. Statistical Analysis

Results were expressed as mean ± SD for continuous variables or frequencies (percentages) for categorical variables. We also reported the 95% confidence interval (95% CI) for the results. A subgroup analysis according to gender was performed in this study. The difference in the score of home-based physical environmental characteristics and home-based social environmental characteristics for boys and girls were examined using the one-way analysis of variance (ANOVA). Due to the non-normal distribution of PA variables, the difference in the volume of different physical behaviors was examined using the Kruskal–Wallis ANOVA. The multiple linear regression analysis in the adjusted model was used to evaluate the influence of different home-based PA environmental domains (PA availability, PA accessibility, Parental role-modeling of PA, and Parental policies around PA) on different physical behaviors (SB, LPA, and MVPA) of adolescents (boys and girls). The model was adjusted for age, height, body mass, parental education, number of siblings, and family monthly outcome. The level of the significance was set at *p* < 0.05. In this study, we also indicated the significance level at *p* < 0.01. The statistical analyses were implemented by using SPSS (Version 22, Chicago, IL, USA).

## 3. Results

### 3.1. Demographic Analysis

The descriptive characteristics of 544 participants, including age (yrs), height (cm), body mass (kg), parental education level, parental age (yrs), number of siblings, and family monthly outcome (RMB, Chinese yuan) are shown in Table 1. The education level of the most adolescents’ father is bachelor degree and that of the most adolescents’ mother is senior high school (and below); the majority of adolescents’ family monthly outcome is 10,000–20,000 RMB.

### 3.2. Gender Difference in Physical Behaviors and Home-Based Environmental Characteristics

**Figure 1:**
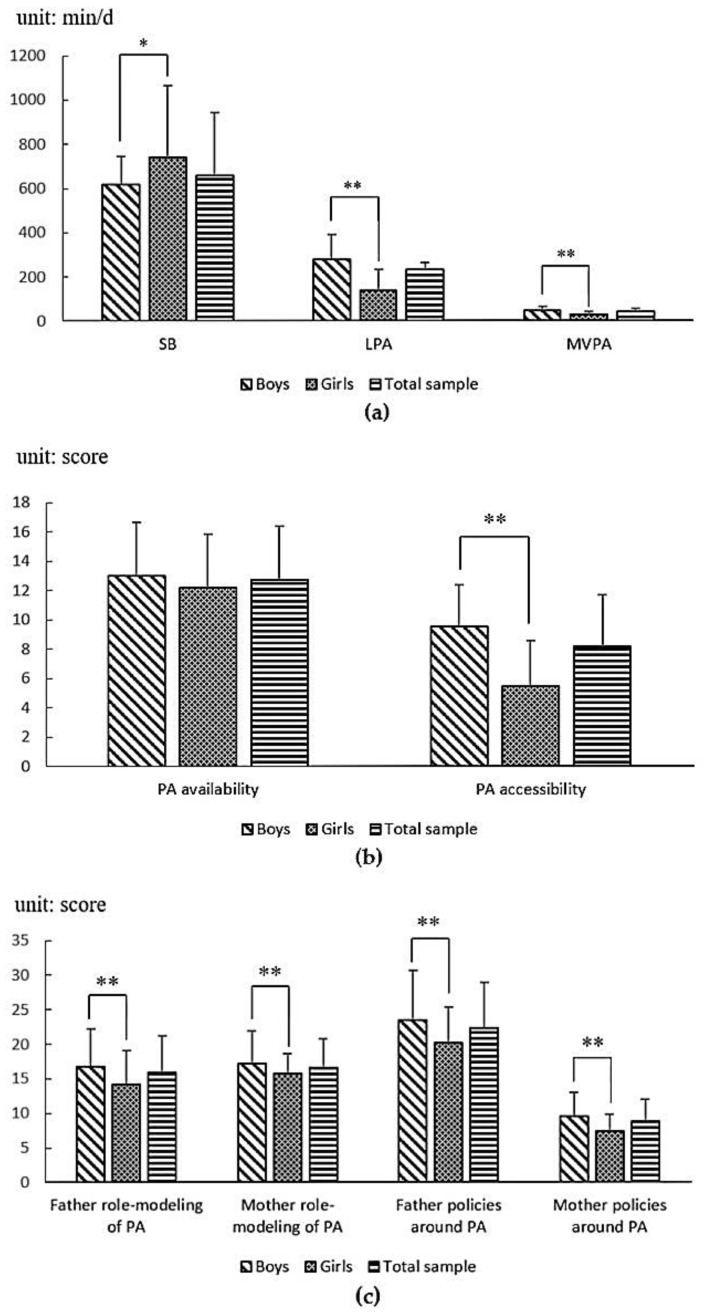
The gender difference in (**a**) physical activity, (**b**) home-based physical environmental characteristics, and (**c**) home-based social environmental characteristics. Abbreviations: SB, sedentary behavior; LPA, light-intensity physical activity; MVPA, moderate-vigorous-intensity physical activity; PA, physical activity; min/d, minutes per day. * Significant difference between boys and girls (*p* < 0.05). ** Significant difference between boys and girls (*p* < 0.01).

The gender difference in (a) physical behaviors, (b) home-based physical environmental characteristics, and (c) home-based social environmental characteristics of 544 adolescents are illustrated in Figure 1. For the PA level, whether it is LPA or MVPA, the weekly participation time of boys (LPA: 280.55 ± 113.07, MVPA: 51.01 ± 18.14) is significantly higher than that of girls (LPA: 139.68 ± 94.49, MVPA: 30.93 ± 12.02, *p* < 0.01). In addition, it also shows significant difference in weekly time of SB between boys and girls; girls’ weekly SB time (741.77 ± 324.88) is much more than that of boys (617.54 ± 128.46, *p* < 0.05). For the home-based physical environment related to PA, the significant difference between boys and girls occurs in the PA accessibility (boys: 9.57 ± 2.84, girls: 5.48 ± 3.14, *p* < 0.01), but not in PA availability (boys: 13.01 ± 3.63, girls: 12.19 ± 3.66, *p* > 0.05). For the home-based social environment related to PA, whether it is father’s role-modeling of PA, mother’s role-modeling of PA, father policies around PA, or mother policies around PA, the score of them in boys (father’s role-modeling of PA: 16.85 ± 5.37, mother’s role-modeling of PA: 17.17 ± 4.76, father policies around PA: 23.52 ± 7.20, mother policies around PA: 9.65 ± 3.38) are significantly higher than those in girls (father’s role-modeling of PA: 14.23 ± 4.89, mother’s role-modeling of PA: 15.71 ± 2.88, father policies around PA: 20.19 ± 5.15, mother policies around PA: 7.38 ± 2.52, *p* < 0.01).

### 3.3. Association between Home-Based Environment and Different Intensity of Physical Activity of Adolescents

Table 2 shows the model for each home-based PA environmental factor adjusted for age, height, body mass, parental education, number of siblings, and family monthly outcome. For the SB, the PA availability (*p* < 0.01), father’s role-modeling of PA (*p* < 0.05), mother’s role-modeling of PA (*p* < 0.01), and mother policies around PA (*p* < 0.05) are significantly negative associated with boy’s weekly SB time, while the PA availability (*p* < 0.01), father’s role-modeling of PA (*p* < 0.01), father policies around PA (*p* < 0.05), and mother’s role-modeling of PA (*p* < 0.01) are significantly negative associated with girl’s weekly SB time. In addition, the model fit of girls (R^2^: 0.44, adjusted R^2^: 0.40) is better than that of boys (R^2^: 0.33, adjusted R^2^: 0.21).

For the LPA, in boys, the PA availability (*p* < 0.01), father’s role-modeling of PA (*p* < 0.05), mother’s role-modeling of PA (*p* < 0.05), and mother policies around PA (*p* < 0.01) have significant positive correlation with the weekly LPA time. In girls, the PA availability (*p* < 0.01), PA accessibility (*p* < 0.05), father policies around PA (*p* < 0.05), and mother’s role-modeling of PA (*p* < 0.01) have significant positive correlation with the weekly LPA time. In addition, the model fit of girls (R^2^: 0.40, adjusted R^2^: 0.35) is better than that of boys (R^2^: 0.26, adjusted R^2^: 0.16).

For the MVPA, boys’ weekly MVPA time is significantly positive related to the PA availability (*p* < 0.05), PA accessibility (*p* < 0.05), father’s role-modeling of PA (*p* < 0.05), mother’s role-modeling of PA (*p* < 0.01), and mother policies around PA (*p* < 0.01). In girls, the weekly MVPA time are significantly positive related to the PA availability (*p* < 0.01), PA accessibility (*p* < 0.05), father’s role-modeling of PA (*p* < 0.05), father policies around PA (*p* < 0.05), and mother’s role-modeling of PA (*p* < 0.01). The model fit of boys (R^2^: 0.31, adjusted R^2^: 0.26), better than that of girls (R^2^: 0.25, adjusted R^2^: 0.20), is an exception.

## 4. Discussion

To the best of our knowledge, this is the first study demonstrating the gender difference in different physical behaviors (SB, LPA, MVPA), the home-based physical environmental characteristics in different domains (PA availability, PA accessibility), and the social environmental characteristics in different domains (father’s role-modeling of PA, father policies around PA, mother’s role-modeling of PA, mother policies around PA) of Chinese adolescents. Our findings on the association between different domains of home-based PA environment and different physical behaviors in teenagers of different genders can provide evidence for the home-based interventions and guidelines on promoting Chinese adolescents’ PA.

The results concerning gender difference in different physical behaviors in our study indicated that the adolescent boys were more active in LPA and MVPA than adolescent girls, and they spent less time every week in SB than girls. The findings are consistent with the results in many previous studies from Japan [37], USA [38], Canada [39], France [40], Germany [41], and England [42]. One study investigated the PA volume of adolescents in 41 countries and found that 28% boys reached the WHO recommended PA volume for adolescents, which suggested that adolescents should perform at least 60 min of MVPA every day, while only 19% girls met the PA recommendation [43]. The gender difference in different intensity of PA might be attributed to the biological factors and sociocultural environment [44,45]. The subjects participating in our study were from 12 to 18 years old, which is adolescence, identified as the transition period from childhood to adulthood. In this period, the difference in body composition between boys and girls is increasing. For example, the muscle strength and muscle mass become greater in boys in youth due to the difference in the biochemical properties and biological structure of the muscle cells caused by male sex hormones [46]. For girls, the PA behavior is more easily affected by the sociocultural environment [47], such as less encouragement of sports participation by social subjective norms [48]. Furthermore, in our study, the time spent sedentary, at 617.54 min/d (minutes per day) and 741.77 min/d in boys and girls, respectively, is not optimistic. These outcomes are higher than that in the England Health Survey (509 min/d) [42], Multicenter European Study (540 min/d) [49], and for the US (480 min/d) [50]. Although several studies clarified that the detrimental effect of SB was independent of PA level [51,52], it was also proved that excessive sedentary time may weaken the beneficial effects of PA [53,54]. There is a study indicating that more MVPA with concomitant higher level of LPA may result in less SB; to be specific, every 6 and 23 min more of MVPA and LPA can reduce 30 min SB [55]. This suggests that both LPA and MVPA can reduce SB, and the higher PA level can not only produce positive effects by itself, but also decreases the harm of SB.

Our study also found that whether it was the PA accessibility in home-based physical environment, the father’s/mother’s role-modeling of PA, or the father/mother policies around PA, the scores of these in boys were higher than those in girls. This may be one potential reason for the significant gender difference in SB, LPA, and MVPA, which was also confirmed in the regression analysis of our study.

In terms of the influence of home-based physical environment on various physical behavior (SB, LPA, MVPA), we can observe a negative association between physical environmental characteristics and SB, and a positive association between physical environment and LPA or MVPA. Compared with PA accessibility, PA availability can significantly promote more kinds of physical behaviors. In addition, the promotion effect of PA availability on various physical behaviors of adolescent girls is greater than that of boys. For the SB, when the score of PA availability increases one point, the SB time decreases 43.17 min/week in boys but 244.24 min/week in girls. For the LPA, boys spend an additional 13.06 min/week for every one additional point of PA availability, while girls’ LPA can increase 18.14 min/week if the score of PA availability increases one point. There is also a similar gender difference on the effect of PA availability on MVPA, one more point of PA availability will lead to an increase of 47.49 min/week of MVPA in boys, but an increase of 69.34 min/week of MVPA in girls. Based on this finding, further home-based PA improvement strategies on girls with insufficient PA and excessive SB can focus more on increasing the presence of PA equipment in the family environment. It is consistent with the result of Dunton and colleague’s study that adolescent girls’ physical behaviors are significant correlated to the number and diversity of sports instruments [31]. In addition, several previous studies support our findings about the important role of home-based physical environmental characteristics on different physical behaviors of adolescents [56], but less research clarified the gender difference. For example, Tandon et al. illustrated that the presence of a basketball hoop at or around the home presented the strongest relationship with young people’s SB and MVPA measured by objective instruments [15]. One interesting meta-analysis [56] raised an opinion that PA equipment at home might reduce SB time through providing the possibility of alternative LPA rather than MVPA. In our study, however, we find that, whether in boys or girls, the PA availability had significant positive association with both LPA and MVPA, and it had a significant negative association with SB. Therefore, we can infer that the home physical environment has the positive promoting impact on various physical behaviors, even MVPA.

For the home social environment, a number of studies proved that there is significant correlation between the PA of adolescents and the home-based social environment. Several researchers objectively measured the PA of adolescents and their parents by accelerometer and reported that parents’ MVPA were positively related to their children’s MVPA [57,58]. In addition, the adolescents in families where parents are willing to exercise with their children and support their children’s participation in PA are likely to perform more active physical behaviors than those in families where parents are unwilling to spend time exercising with their children and encouraging their children to conduct PA [59,60]. These results of previous studies support the findings about the association between home social environment and teenagers’ PA in our study. Furthermore, there is another very interesting finding in our study. In the regression analysis, we found that the PA role-modeling of the same-sex parent–child dyads could produce more positive influence on adolescents’ various physical behaviors than that of opposite-sex parent–child dyads. To be specific, for boys, one score of father’s role-modeling of PA can reduce an additional 42.75 min/week of SB, while one score of mother’s role-modeling of PA can only reduce an additional 24.73 min/week of SB. Every one score of father’s role-modeling of PA for boys can increase an additional 1.8 min/week of LPA and 41.33 min/week of MVPA, which is more than the promoting effect of mother’s role-modeling of PA on boys’ LPA (1.39 min/week) and MVPA (22.96 min/week), respectively. A similar trend also occurs in girls. For girls, mother’s role-modeling of PA can produce more positive impact on SB, LPA, and MVPA than the beneficial effect of father’s role-modeling of PA. This may be explained by the gender-role identity in adolescence; for example, some studies implicated an association between adolescent girls’ body image and their mothers’ body image [61,62], because mothers serve as vital role models and a source of information and guidance in girls’ developmental stage [63], and it would seem innately probable to attain a likeness, including physical behavior, to the girl’s biological mother. Except that, the effect of parents’ policies around PA also shows the gender difference in adolescents. Interestingly, whether for boys or girls, parents’ encouragement and supportive policies related to PA in opposite-sex parent–child dyads present significant association with adolescents’ different physical behaviors (SB, LPA, and MVPA); however, the association between PA policies of parents and adolescents’ PA in same-sex parent–child dyads is not significant. This result is inconsistent with one of the few related studies—Savage et al. illustrated that fathers’ and mothers’ encouragement was associated with their daughters’ PA, but not their sons’ PA [64]. The initial reason for this contradiction is unclear; it reminds us further study should focus more on investigating the moderating effect of sex between opposite-sex parent–child dyads and same-sex parent–child dyads.

The major strength of this study is that we reveal the gender difference in association between different domains of home-based environmental characteristics and different physical behaviors. Most importantly, our study found the PA availability, the parents’ role-modeling of PA in same-sex parent–child dyads, and the parents’ policies around PA in opposite-sex parent–child dyads could significantly improve adolescents’ healthy physical behaviors, which could be applied to develop the promotion strategies related to home-based environment for Chinese adolescents’ physical behaviors. On the other hand, there are several limitations in our study. First, the cross-sectional design failed to draw a rational causal relationship between home environment and adolescents’ physical behaviors; additional longitudinal research is needed to prove our results. Second, for investigating a public health problem, the sample size in our study is not enough. In China, the youth population base is very large—the subjects in our study may not represent overall the characteristics of home-based environment and physical behaviors of adolescents in China. Third, the confounding factors involved in the model are not adequately controlled, which may lead to biased results. For example, the building environment of the home and selected transportation can affect the PA volume of adolescents. In further studies, we should conduct a comprehensive survey on the influential factors of adolescents’ physical behaviors, which would provide results closer to the real situation by controlling more confounding factors. Fourth, we did not test the reliability and validity of Gattshall’s home-based PA environment questionnaire based on the Chinese adolescents’ parents, which may also cause bias in our results.

## 5. Conclusions

Collectively, there is significant gender difference in adolescents’ physical behaviors and home-based environmental characteristics, as well as in association between adolescents’ physical behaviors and their home-based environment. Both of the home physical environment and social environment can promote adolescents’ healthy physical behaviors, especially the PA availability, the parents’ role-modeling of PA in same-sex parent–child dyads, and the parents’ policies around PA in opposite-sex parent–child dyads. These findings may provide scientific evidence for parents to manage more active home-based PA environment, which can be helpful for dealing with the public health issues of insufficient PA among adolescents.

## Figures and Tables

**Table 1:** Descriptive characteristics of 545 adolescents.

	Total Sample	Boys	Girls
*n*	544	358	186
Mean (SD) or *n* (%) ^a^			
Adolescent			
age (yrs)	15.05 (2.29)	15.38 (1.62)	14.41 (3.11)
height (cm)	171.51 (4.17)	176.30 (5.22)	162.31 (3.90)
body mass (kg)	66.89 (11.73)	71.62 (10.07)	57.79 (12.04)
Parent			
Parental education (father)			
senior high school (and below)	165 (30.28%)	103 (28.77%)	62 (33.33%)
junior college	118 (21.65%)	74 (20.67%)	44 (23.66%)
bachelor	199 (36.51%)	140 (39.11%)	59 (31.72%)
master	41 (7.52%)	25 (6.98%)	16 (8.60%)
doctor	21 (3.86%)	15 (4.19%)	6 (3.23%)
Parental education (mother)			
senior high school (and below)	168 (30.83%)	105 (29.33%)	63 (33.87%)
junior college	163 (29.92%)	106 (29.61%)	57 (30.65%)
bachelor	161 (29.54%)	109 (30.45%)	52 (27.96%)
master	46 (8.44%)	33 (9.22%)	13 (6.99%)
doctor	6 (1.10%)	4 (1.12%)	2 (1.08%)
Parental age			
father age (yrs)	43.80 (4.90)	44.48 (4.52)	42.48 (5.30)
mother age (yrs)	42.12 (3.89)	42.59 (3.92)	41.20 (3.68)
Family			
Number of siblings	1.29 (0.49)	1.25 (0.46)	1.37 (0.52)
Family monthly outcome (RMB)			
less than 1500	3 (0.55%)	3 (0.84%)	0 (0.00%)
1500–3000	23 (4.23%)	16 (4.47%)	7 (3.76%)
3001–6000	112 (20.59%)	69 (19.27%)	43 (23.12%)
6001–10,000	119 (21.88%)	80 (22.35%)	39 (20.97%)
10,000–20,000	148 (27.21%)	105 (29.33%)	43 (23.12%)
20,000–50,000	112 (20.59%)	69 (19.27%)	43 (23.12%)
more than 50,000	27 (4.96%)	16 (4.47%)	11 (5.91%)

Abbreviations: yrs, years; cm, centimeters; kg, kilograms; RMB, Chinese yuan. ^a^ Frequencies are the percentages shown in the column.

**Table 2:** The association between home-based environment and adolescents’ weekly physical activity in different intensity.

Variables	Boys				Girls	
	B(SE)	95%CI	β	B(SE)	95%CI	β
SB						
The SB volume (min/wk)	4322.77 ± 899.20 ^^^			5192.41 ± 2274.16		
PA availability	−43.17 (44.40)	(−66.75, −19.59)	−0.18 **	−244.24 (67.08)	(−356.98, −91.49)	−0.28 **
PA accessibility	64.39 (50.91)	(38.49, 90.29)	0.22	88.93 (52.39)	(−14.75, 192.61)	−62.49
Father’s role−modeling of PA	−42.75 (43.77)	(−45.84, −39.66)	−0.17 *	−364.08 (74.94)	(−512.39, −215.78)	−0.4 **
Father policies around PA	18.88 (58.79)	(7.68, 30.08)	0.11	−276.19 (1115.45)	(−504.66, −47.73)	−0.36 *
Mother’s role−modeling of PA	−24.73 (64.89)	(−37.61, −11.85)	−0.12 **	−452.24 (121.24)	(−692.16, −212.32)	−0.87 **
Mother policies around PA	−30.60 (67.58)	(−53.19, −8.01)	−0.10 *	63.86 (113.50)	(−160.75, 288.46)	0.06
R^2^ (adjusted R^2^)	0.33 (0.21)			0.44 (0.40)		
LPA						
The LPA volume (min/wk)	1963.84 ± 791.49 ^^^^			977.73 ± 661.43		
PA availability	13.06 (10.50)	(8.15, 17.97)	0.21 **	18.14 (3.74)	(10.75, 25.54)	0.38 **
PA accessibility	16.89 (12.03)	(7.42, 26.36)	0.23	10.98 (4.78)	(1.51, 20.44)	0.20 *
Father’s role-modeling of PA	1.80 (2.34)	(0.03, 3.57)	0.04 *	−8.43 (8.65)	(−25.54, 8.68)	−0.24
Father policies around PA	−12.56 (13.90)	(−40.65, 15.52)	−0.30	7.76 (8.23)	(6.99, 8.53)	0.23 *
Mother’s role-modeling of PA	1.39 (1.35)	(0.87, 1.91)	0.02 *	21.98 (5.35)	(11.40, 32.56)	4.11 **
Mother policies around PA	11.86 (5.98)	(4.72, 19.00)	0.16 **	12.95 (8.10)	(−3.07, 28.97)	1.60
R^2^ (adjusted R^2^)	0.26 (0.16)			0.40 (0.35)		
MVPA						
The MVPA volume (min/wk)	357.07 ± 126.95 ^^^^			216.51 ± 84.22		
PA availability	47.49 (40.04)	(6.22, 88.76)	0.21 *	69.34 (14.86)	(39.94, 98.74)	0.41 **
PA accessibility	30.07 (24.92)	(7.14, 53.00)	0.16 *	20.10 (19.02)	(17.54, 22.66)	0.11 *
Father’s role−modeling of PA	41.33 (34.43)	(8.25, 74.41)	0.22 *	13.02 (34.38)	(−55.01, 81.05)	0.09 *
Father policies around PA	6.29 (6.24)	(−3.34, 15.92)	0.05	15.66 (12.74)	(9.12, 22.20)	0.10 *
Mother’s role−modeling of PA	22.96 (11.05)	(14.06, 31.86)	0.14 **	59.68 (21.25)	(17.63, 101.73)	0.28 **
Mother policies around PA	18.72 (13.16)	(11.90, 25.54)	0.08 **	35.17 (32.18)	(3.52, 66.82)	0.14
R^2^ (adjusted R^2^)	0.31 (0.26)			0.25 (0.20)		

Abbreviations: SB, sedentary behavior; LPA, light−intensity physical activity; MVPA, moderate-vigorous-intensity physical activity; B: nonstandardized regression coefficient; SE: standard error; CI: confidence interval; β: standardized regression coefficient; R^2^, R square. * Significant difference in standardized regression coefficient of the model (*p* < 0.05). ** Significant difference in standardized regression coefficient of the model (*p* < 0.01). ^^^ Significant difference in weekly physical activity volume between boys and girls (*p* < 0.05). ^^^^ Significant difference in weekly physical activity volume between boys and girls (*p* < 0.01).

## Appendix A

**Table A1:** The specific items of each home-based PA environment domain (based on Gattshall’s home-based PA environment questionnaire) [32].

Home-Based PA Environment	Home-Based PA Environment Domain	Items or Questions
Physical environment	PA availability	Inside playroom
		Exercise room
		Dance room, Yoga room, or Taekwondo room
		Driveway
		Play area/yard
		Exercise equipment in TV area
		Space to play in TV area
		Swing set
		Bicycle
		Rollerblades/skates
		Skateboard/scooter
		Jump rope
		Hiking shoes
		Running shoes
		Basketball hoop
		Baseball equipment
		Racket
		Sports application (APP) in digital equipment
		Balls
		Pedometer
		Winter sports equipment
		Other physically active toys
	PA accessibility	How many of your child’s active toys are in working condition
		How many of your child’s active toys are stored in area child uses them
		How many of your child’s active toys does child need help getting out *
		How many of your child’s active toys are stored out of sight *
Social environment	Parental role-modeling of PA	Your child sees you being physically active
		Your child sees you doing house/yard work
		Your child sees you use PA as relaxation
		Your child sees you on the computer *
		Your child sees you watching TV/movies *
		Your child hears you talk about sports or PA
		Your child hears you say you are too tired to be physically active *
		How often are you physically active with your child
	Parental policies around PA	How often do you encourage your child to be physical active
		How often do you transport your child for PA
		How often do you send your child outside to play
		How often do you give your child PA options
		How often do you praise your child for being physically active

Abbreviations: PA, physical activity; * represents the items that are reverse coded.
